# Socioeconomic Burden of Influenza in the Republic of Korea, 2007–2010

**DOI:** 10.1371/journal.pone.0084121

**Published:** 2013-12-27

**Authors:** Mina Suh, Dae Ryong Kang, Dong Han Lee, Yoon Jung Choi, Byongho Tchoe, Chung Mo Nam, Hyung Jung Kim, Jong Koo Lee, Byung Yool Jun, Yoosik Youm, Gwi-Nam Bae, Tae Yong Lee, Moon Shik Kim, Dong Chun Shin, Changsoo Kim

**Affiliations:** 1 National Cancer Control Institute, National Cancer Center, Goyang, Korea; 2 Yonsei University College of Medicine, Seoul, Korea; 3 Korea Centers for Disease Control and Prevention, Osong, Korea; 4 Helath Insurance Review & Assessment Service, Seoul, Korea; 5 Seoul National University Hospital, Seoul, Korea; 6 Department of Sociology, Yonsei University, Seoul, Korea; 7 Environmental Sensor System Research Center, Korea Institute of Science and Technology, Seoul, Korea; 8 Department of Preventive Medicine and Public Health, Chungnam National University School of Medicine, Daejeon, Korea; 9 School of Public Health, Ajou University, Suwon, Korea; University of Calgary & ProvLab Alberta, Canada

## Abstract

**Background:**

Although the socioeconomic burden of 2009 pandemic influenza A (H1N1) was considerable, no reliable estimates have been reported. Our aim was to compared medical costs and socioeconomic burden resulting from pandemic influenza A (H1N1) 2009 with that of previous seasonal influenza.

**Methods:**

We estimated the medical costs and socioeconomic burden of influenza from May 2007 to April 2010. We used representative national data sources(data from the Health Insurance Review Agency, the National Health Insurance Corporation, the Korea Centers for Disease Control and Prevention, and the Korean National Statistics Office) including medical utilization, prescription of antivirals, and vaccination. Uncertainty of data was explored through sensitivity analysis using Monte Carlo simulation.

**Results:**

Compared with the seasonal influenza, total medical costs (US$291.7 million) associated with pandemic (H1N1) 2009 increased more than 37-fold. Compared with the 2007–2008 season, outpatient diagnostic costs (US$135.3 million) were 773 times higher in the 2009–2010 season, and the mean diagnostic cost per outpatient visit was 58.8 times higher. Total socioeconomic burden of pandemic (H1N1) 2009 was estimated at US$1581.3 million (10%–90%: US$1436.0–1808.3 million) and those of seasonal influenza was estimated at US$44.7 million (10%–90%: US$32.4–57.9 million) in 2007–2008 season and US$42.3 million (10%–90%: US$31.5–53.8 million) in 2008–2009 season. Indirect costs accounted for 56.0% of total costs in pandemic (H1N1) 2009, and 66.48–68.09% in seasonal influenza. The largest contributors to total burden were productivity losses of caregiver in pandemic (H1N1) 2009, and productivity losses due to morbidity of outpatient in seasonal influenza.

**Conclusions:**

In the Republic of Korea, socioeconomic burden of pandemic (H1N1) 2009 were considerably higher than burden of the previous two influenza seasons, primarily because of high diagnostic costs and longer sick leave.

## Introduction

After the first cases of swine-origin influenza A were described in Mexico and the United States in April 2009, pandemic influenza A (H1N1) 2009 spread throughout the world. [1], [2] The first case of pandemic (H1N1) 2009 in the Republic of Korea (ROK) was identified in a person returning from Mexico on May 2, 2009, and virus activity subsequently increased rapidly. [3]–[6].

Because of the high rates of incidence, the medical costs of pandemic (H1N1) 2009 have been considerable. [7]–[9] In order to prevent the spread of pandemic (H1N1) 2009, the government was not performed only the health policy, such as quarantine, isolation, and campaign for hygiene, but also the government was responsible for stockpiling antivirals and developing and producing vaccines. [3], [10] Pandemic (H1N1) 2009 would become a significant burden in both healthcare and socioeconomic system.

On the other hands, the government was conducted surveillance system for investment the scale occurred influenza. However, the surveillance may have been under-reporting, because the surveillance was included serological confirmed patients. Therefore, efforts are needed to estimate the exact socioeconomic burden of pandemic (H1N1) 2009.

Although several studies have been conducted to estimate disease the burden or medical costs of pandemic (H1N1) 2009, these studies focused on disease severity, effective vaccination, or community mitigation strategies. [11]–[16] Due to limitations of representative national statistics, estimation on economic impact of pandemic (H1N1) 2009 has not been conducted.

The purpose of this study was to determine the socioeconomic burden of seasonal influenza (2007–2008 [May 2007 to April 2008] and 2008–2009 seasons [May 2008 to April 2009]) and pandemic (H1N1) 2009 (2009–2010 influenza season [May 2009 to April 2010]) in the ROK and to compare medical costs and socioeconomic burden of pandemic (H1N1) 2009 with those of seasonal influenza.

## Methods

### Data Sources

#### Medical Care Utilization Data

Medical care utilization data (e.g., date clinic/hospital visit, medical service, and cost per service) were obtained from the Health Insurance Review & Assessment Service (HIRA). (Table 1) All legal residents of the ROK are covered by the National Health Insurance program, which uses a fee-for-service payment system to reimburse healthcare providers. [17] The Korean government regulates these fees, and HIRA has the authority to review healthcare insurance claims and assess healthcare quality. We selected all insurance claims in which influenza was diagnosed (International Classification of Diseases, 10th Revision, Clinical Modification [ICD-10-CM] codes: J09–J11) between May 2007 and April 2010.

**Table 1:** Parameter and data sources.

	parameter	Distribution of assumption factors*	Data source	
			Seasonal influenza (2007–2009 Seasons)	H1N1 Influenza 2009 (2009–2010 Season)
**Direct costs**				
Direct medical costs				
Medical costs of inpatient and outpatient	Total medical cost		HIRA data	HIRA data
Stockpile antivirals	Total cost of antiviral		-	NHIC data
Direct non-medical costs				
Transport costs of inpatient	Number of visits to inpatient		HIRA data	HIRA data
	Return fare	Normal (Mean = 19.2, SD = 1.92)	KNHNES	KNHNES
Transport costs of outpatient	Number of visits to outpatient		HIRA data	HIRA data
	Return fare	Normal (Mean = 15.5, SD = 1.55)	KNHNES	KNHNES
**Indirect costs**				
Productivity losses due to morbidity of inpatient	Number of visits to inpatient & Duration of hospitalization		HIRA data	HIRA data
	Average daily earnings & Employment-population ratio		KOSIS data	KOSIS data
Productivity losses due to morbidity of outpatient	Number of visits to outpatient		HIRA data	HIRA data
	Duration of sick leave	Seasonal influenza: Uniform (Range: 0.5–4.5); H1N1 Influenza 2009: Negative binomial (Probability = 0.7166, Shape = 5)	Literature review	Mailing survey
	Average daily earnings & Employment-population ratio		KOSIS data	KOSIS data
Productivity losses of caregiver	Duration of sick leave	Seasonal influenza: Uniform (Range: 0.5–4.5); H1N1 Influenza 2009: Negative binomial (Probability = 0.7166, Shape = 5)	Literature review	Mailing survey
	Female average daily earnings & Employment-population ratio		KOSIS data	KOSIS data
Productivity losses due to premature mortality	Mortality data		KOSIS data	KCDC surveillance data
	Life expectancy & Average annual earnings		KOSIS data	KOSIS data
**Prevention strategy**				
Execution of the budget	Execution of the budget regarding pandemic (H1N1) 2009		-	KNAB
Protective equipment	Price of prevention equipment	Uniform (Range: 0.47–4.7)	-	Literature review
	Probability of purchasing	Normal (Mean = 0.325, SD = 0.03)	-	Literature review

*Uncertainty of the data was explored through probabilistic sensitivity analysis using Monte Carlo simulation.

#### Monitoring of stockpiled antivirals

Before 2009–2010 season, because few antivirals for influenza were on the market in the ROK due to rare prescriptions, almost all antivirals for pandemic (H1N1) 2009 were stockpiled and managed by the government in 2009–2010 season. Since August 21, 2009, the Korea Centers for Disease Control and Prevention (KCDC) and National Health Insurance Corporation have monitored daily prescription of antivirals for influenza. [5].

#### Mortality Data

Death certificate data from the Korean Statistical Information Service (KOSIS) were used to determine influenza mortality (ICD-10-CM: J09–J11) for seasonal influenza during 2007–2009. [18] The KCDC began active surveillance of pandemic (H1N1) 2009-related mortality from August 15, 2009 (date of the first fatality), and a fatal case was defined as a person with pandemic (H1N1) 2009, confirmed by ante-mortem or post-mortem specimens, who died from a clinically compatible illness or complications attributable to that infection. In 2009–2010 season, we used surveillance data from KCDC from August 15, 2009 until April 30, 2010.

### Estimating Cost of Illness

The socioeconomic burden of influenza was estimated as direct costs, indirect costs, and costs associated with prevention strategies. To compare the socioeconomic burden of seasonal influenza in the 2007–2008 and 2008–2009 seasons with that of pandemic (H1N1) 2009, all resource utilization estimates were obtained for 2009 and expressed in 2009 United States dollars (US$) using the 2009 average exchange rate (US$1 = 1276.4 Korean won; Bank of Korea).

#### Direct Costs

Direct healthcare costs consisted of medical services and medication. Total medical costs associated with influenza(ICD-10-CM codes: J09–J11) were obtained by adding costs reported by all clinics and hospitals to HIRA for each season. Non-stockpile antivirals and other drug costs were included in medication costs reported by HIRA. Because stockpiled antivirals were provided for free only in the 2009–2010 season, cost of stockpiled antivirals was estimated as the cost of each drug multiplied by the total number of prescriptions.

Direct non-healthcare costs consisted of transport costs related to clinic or hospital visits. Transport costs were estimated by multiplying the total number of clinic visits by the return fare, reported by the 2005 Korean National Health and Nutrition Examination Survey. [19] To estimate the cost of return fares, the healthcare component of the Korean Consumer Price Index was used to adjust costs to the 2009 values.

#### Indirect Costs

Indirect costs resulting from productivity losses were estimated as: 1) productivity losses due to morbidity, 2) productivity losses of caregiver, and 3) premature mortality resulting from influenza. Indirect costs were estimated using a human capital approach by multiplying expected mean earnings by time lost at work and adjusted by the employment-population ratio by matching gender and age.

Productivity losses of adults (20–64 years old) were estimated from the average earnings in the general population (adjusted by the employment–population ratio) by matching gender and age. [18] For inpatients, we multiplied average daily earnings by hospitalization day. For outpatients, we multiplied average daily earnings by sick leave duration based on the first outpatient visit. Subsequent outpatient visits were assumed to be follow-up appointments for influenza, and work loss was estimated as a half day. For calculating the outpatients productivity losses, sick leave of outpatients (range: 0.5–4.5 days) was assumed according previously published data for the 2007–2008 and 2008–2009 seasons. [20], [21] For the 2009–2010 season, we surveyed patients with laboratory-confirmed pandemic (H1N1) 2009 between August 2009 and February 2010 in four university-based quarantine hospitals (representing four provinces in the ROK). The survey asked, “How many days of sick leave did you take to stay home with influenza?” We mailed questionnaires to 10,833 patients in February and May 2010, and 2,166 patients (20.0%) responded. Median sick leave of patients was 7 days (range: 1–28).

Unless they were admitted to the hospital, we assumed that children ≤19 years and adults ≥65 years required care from a family member, and that most caregivers were women. Caregiver productivity losses were calculated by multiplying the time required to care for a sick family member by average daily earnings.

Lost earnings due to premature mortality were calculated from the number of influenza-related deaths and the annual earnings of those patients. Only patients <65 years old (standard retirement age) were included in this analysis. The expected future loss of earnings due to premature mortality was adjusted using a 5% discount rate. Life expectancy, average annual earnings, and employment–population ratio by age and gender were based on 2009 general population data from the KOSIS. [18].

#### Prevention Strategy

Costs associated with prevention strategies (national budget for influenza prevention and use of protective equipment) were estimated only for the 2009–2010 season, because complete data were not available for the other seasons. Data for execution of the budget regarding pandemic (H1N1) 2009 (quarantine facilities, vaccine development, vaccine/antiviral stockpiles) were obtained from the Korean National Assembly Budget office. [22] Direct costs consisted of the cost of stockpiled antivirals based on the actual usage; costs associated with used stockpiled antivirals were excluded for estimating of the costs associated with prevention strategies. The cost of protective equipment (e.g., masks, hand sanitizers) was calculated by multiplying purchase price by the probability of purchasing (estimated as 32.5%), based on a survey. [23] Direct and indirect influenza-related costs were based on the target population (patients), however prevention costs were based on the entire population.

### Statistical Analysis

Total costs were defined as the sum of direct costs, indirect costs, and costs associated with prevention strategies. Data were compared by chi-square test or one-way analysis of variance to determine differences in medical costs across seasons. While available national data such as medical costs and cost of antiviral was used as fixed values, some data such as transport costs and duration of sick leave, used as assumed value. Therefore, uncertainty of the assumed data was explored through probabilistic sensitivity analysis (1,000 independent simulation trials) using Monte Carlo simulation (Oracle Crystal Ball, version 11.1.1.30, Oracle Corporation). the Monte Carlo simulation assumed normal distribution for transport costs (mean ± standard deviation; inpatients: 19.16±1.916, outpatients: 15.47±1.547), uniform distribution of sick leave days for seasonal influenza (range: 0.5–4.5) in 2007–2008 and 2008–2009 seasons, and negative binomial distribution for sick leave associated with pandemic (H1N1) 2009 (probability  = 0.7166, shape  = 5), which was fitted to questionnaire data. (see Table 1 for more details on parameters and distribution of assumption factor).

### Ethics Statement

This study was approved by the institutional review board of Yonsei University Health System (approval number: 4-2011-0279).

## Results

**Figure 1:**
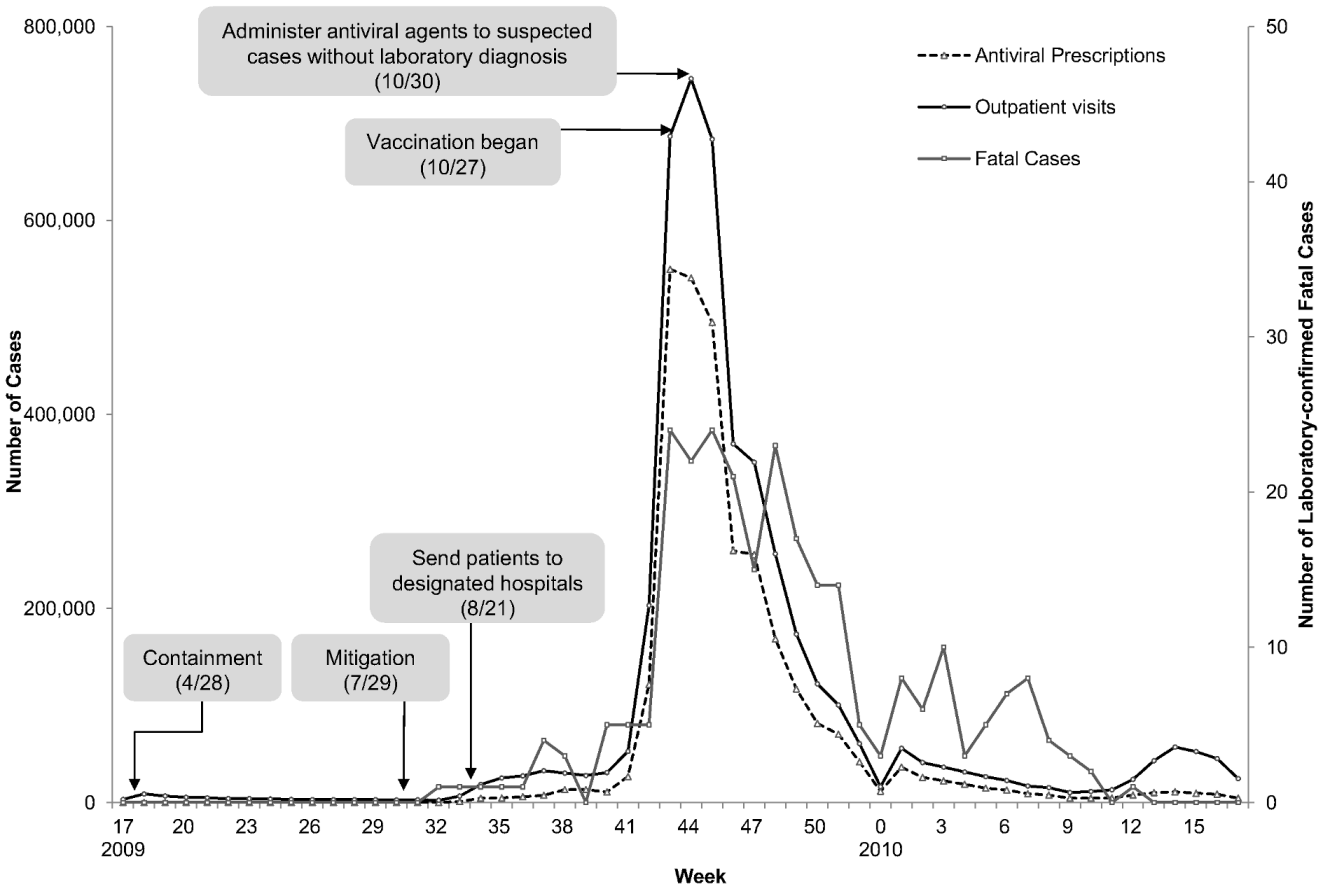
Outbreak of Pandemic Influenza A (H1N1) 2009 and Main Response Strategy in Korea.

During the 2009–2010 season (week 17, 2009 through week 16, 2010), a total of 266 fatal cases were reported (Figure 1). Outpatient visits and antiviral prescriptions peaked at week 43. Vaccinations against pandemic (H1N1) 2009 began on October 27, 2009, when the number of outpatient visits peaked (746,290/week).

Inpatient visits during the 2009–2010 season (123,035) increased 15- to 20-fold compared with two previous seasons, and outpatient visits (4,609,026) increased 10-fold (Table 2). The number of visits per person during the 2009–2010 season was not increased compared with two previous seasons. During the 2009–2010 season, total medical costs of inpatients increased 20-fold and those costs of outpatients increased 50-fold. In both inpatient and outpatient, the medical costs of all subcategory during the 2009–2010 season increased compared with two previous seasons. The proportion of each subcategory among inpatient medical costs was not different among seasons (P-value  = 0.598); whereas, the proportion of each subcategory among outpatient differed significantly (P-value <0.001). The mean cost per visit differed significantly across seasons for both inpatients and outpatients. Among the subcategory of medical cost, the mean diagnostic cost per visit in the 2009–2010 season was significantly increased compared with two previous seasons.

**Table 2:** Number of Visits and Medical Costs for Influenza in the Republic of Korea, 2007–2010.

	2007–2008 Season		2008–2009 Season		2009–2010 Season		P-value*
**Total number of visits**							
Inpatients	6,502		8,775		123,035		
Outpatients	446,713		404,623		4,609,026		
**Number of visits per person** †							
Inpatients	1.07±0.28		1.05±0.22		1.06±0.25		
Outpatients	1.84±2.26		1.75±2.10		1.45±1.07		
**Medical costs** (thousand US$)							
Inpatients							
Total costs	2,740.4	(100.0)	3,799.3	(100.0)	77,004.9	(100.0)	
Consultation costs	1,358.0	(49.6)	1,924.8	(50.7)	34,359.8	(44.6)	0.598
Diagnostic costs	513.1	(18.7)	717.2	(18.9)	20,872.1	(27.1)	
Medication and other costs	869.2	(31.7)	1,157.4	(30.5)	21,773.0	(28.3)	
Outpatients							
Total costs	4,428.9	(100.0)	4,102.7	(100.0)	214,649.0	(100.0)	
Consultation costs	3,897.4	(88.0)	3,544.2	(86.4)	55,542.1	(25.9)	<0.001
Diagnostic costs	175.0	(4.0)	205.0	(5.0)	135,313.9	(63.0)	
Medication and other costs	356.6	(8.1)	353.5	(8.6)	23793.0	(11.1)	
**Costs per visit** (US$)†							
Inpatients							
Total costs	421.4±422.75		432.9±411.83		625.8±800.81		<0.001
Consultation costs	208.8±213.38		219.3±190.71		279.2±356.30		<0.001
Diagnostic costs	78.9±105.86		81.7±103.78		169.6±190.50		<0.001
Medication and other costs	133.7±145.33		131.9±173.53		176.9±347.68		<0.001
Outpatients							
Total costs	9.9±7.53		10.1±9.10		16.6±56.32		<0.001
Consultation costs	8.7±4.16		8.8±3.74		12.0±9.54		<0.001
Diagnostic costs	0.4±3.65		0.5±5.32		29.4±41.64		<0.001
Medication and other costs	0.8±2.35		0.9±3.37		5.2±21.17		<0.001

*P-values for proportional difference of subcategories of medical costs across seasons were determined by chi-square tests, and P-values for mean difference of costs per visit across seasons were determined by analysis of variance.

Results are expressed as mean ± standard deviation.

We estimated the socioeconomic costs of influenza consisted of direct costs (direct medical and non-medical costs), indirect costs (productivity losses of morbidity, caregiver, and premature mortality), and costs associated with prevention strategies (execution of the budget and protective equipment) (Table 3). We varied the return fare for outpatients and inpatients, the duration of sick leave, and the probability of purchasing and the price of preventative equipment. Based on results of the Monte Carlo simulation, the socioeconomic burden for pandemic (H1N1) 2009 was estimated at US$1,581.3 million, and those of seasonal influenza were US$44.7 million in 2007–2008 season and US$42.3 million in 2008–2009 season. Indirect costs were the largest proportion of total costs. In 2009–2010 season, 56.0% of total costs were indirect costs, consisting primarily of productivity losses of caregivers (30.0%) and morbidity (outpatient: 24.3%, inpatient 0.62%). In 2007–2008 and 2008–2009 seasons, the cost of productivity losses due to morbidity was the largest component.

**Table 3:** Estimating Costs of Illness for Pandemic Influenza A (H1N1) 2009 and Seasonal Influenza in the Republic of Korea, 2007–2010.

	2007–2008 Season		2008–2009 Season		2009–2010 Season	
Category	Cost (million US$)	% total costs	Cost (million US$)	% total costs	Cost (million US$)	% total costs
**Direct costs**						
Direct medical costs						
Medical costs of inpatients	2.74	6.16	3.80	8.90	77.00	4.79
Medical costs of outpatients	4.43	9.95	4.10	9.61	214.61	13.37
Stockpile antivirals	NA	NA	NA	NA	41.46	2.58
Direct non-medical costs						
Transport costs of inpatients	0.12 (0.11–0.14)	0.28	0.17(0.15–0.19)	0.39	2.35 (2.04–2.65)	0.15
Transport costs of outpatients	6.91 (5.98–7.84)	15.53	6.23 (5.50–7.01)	14.61	71.16 (61.77–80.78)	4.43
**Indirect costs (Productivity losses)**						
Productivity losses due to morbidity of inpatient	0.60	1.35	0.62	1.46	9.93	0.62
Productivity losses due to morbidity of outpatient	20.10(8.99–32.58)	45.16	18.31(7.86–28.98)	42.92	390.23 (281.16–499.30)	24.30
Productivity losses of caregiver	9.54(4.38–14.42)	21.43	9.32(4.22–14.52)	21.84	480.00(347.75–612.25)	29.89
Productivity losses due to premature mortality	0.07	0.15	0.11	0.26	19.16	1.19
**Prevention strategy**						
Execution of the budget	NA	NA	NA	NA	259.26	16.15
Protective equipment	NA	NA	NA	NA	40.60 (14.50–68.85)	2.53
**Total socioeconomic costs**	44.65 (32.35–57.87)	100.00	42.31 (31.50–53.75)	100.00	1,581.27 (1435.96–1808.33)	100.00

NA: not available Costs are expressed as fixed value or median value (range, 10%–90%).

## Discussion

Inpatient visits, outpatient visits, and total medical costs increased significantly in the 2009–2010 season compared to two previous seasons in the ROK. The 2009–2010 medical costs (US$291.7 million) accounted for 1.24% of the 2009 total national healthcare expenditures (US$23.48 billion). In contrast, 2007–2008 medical costs (US$7.17 million) accounted for only 0.04% of 2007 national healthcare expenditures (US$19.25 billion), and 2008–2009 medical costs (US$7.90 million) were about 0.04% of 2008 national healthcare expenditures (US$20.67 billion). These findings demonstrate the significance of pandemic (H1N1) 2009 as a major public health concern in the ROK. [24].

Compared with the 2007–2008 season, outpatient diagnostic costs were 773 times higher in the 2009–2010 season, and the mean diagnostic cost per outpatient visit was 58.8 times higher. In addition to the increased number of influenza cases, the higher medical costs during the 2009–2010 season may be due to the increased use of diagnostic tests including real-time reverse transcriptase-polymerase chain reaction (RT-PCR). Although laboratory tests can confirm influenza cases, clinical judgment by physicians is important in the identification of influenza. On October 30 (week 43) 2009, the government recommended antivirals administration who met the case definition of influenza-like illness, without laboratory confirmation. However, diagnostic costs did not decrease after that time.

Besides public fears about the pandemic, other factors may be associated with the increased diagnostic testing. First, in the ROK, medical cost consist those covered by insurer and those covered by patients (coinsurance) due to the National Health Insurance program. Before March 2010, the National Health Insurance program covered 40–50% of the cost for RT-PCR testing, suggesting overutilization of these tests because they were relatively inexpensive for the patient. After March 2010(week 11), the patient covered up to 100% of real-time RT-PCR costs, decreasing its proportion of diagnostic costs. Second, most schools and workplaces required negative diagnostic test results for suspected cases during the pandemic, despite government recommendations. Therefore, suspected cases visited clinics and were examined laboratory tests to obtain a medical certificate and not to get treatment. The medical cost breakdown did not differ significantly among age groups, suggesting that age groups not incurred greater diagnostic costs (data not shown).

In this study, total 2009–2010 socioeconomic costs (US$1,581.3 million) accounted for 0.19% of the 2009 gross domestic product (US$832.9 billion). In contrast, total 2007–2008 socioeconomic costs (US$44.7 million) accounted for only 0.005% of the 2008 gross domestic product. In the 2007–2008 and 2008–2009 seasons, indirect costs were the largest proportion of total costs. Specifically, 56.0% of 2009–2010 costs were indirect costs, consisting primarily of productivity losses of caregivers (30.0%) and adult outpatients (24.3%). In the pandemic's early stage, public health officials recommended that students and employees with influenza rest at home for a week, whereas the mean sick leave was <1 to 4.3 days for typical seasonal influenza. [20], [25] This longer sick leave may have contributed to the high indirect costs for pandemic (H1N1) 2009.

The ROK has several national surveillance systems including pneumonia and influenza surveillance and sentinel surveillance for influenza-like illness known as the Korea Influenza Surveillance Scheme. [9] However, the ROK does not have a hospital-based surveillance system for emerging diseases, such as the Emerging Infections Program in the United States, which collects data about susceptibility, clinical course, treatment efficacy, and outcomes. [26] Therefore, little information on these data was available in the early stages of the pandemic in the ROK. Although the severity of pandemic (H1N1) 2009 was similar to that of typical seasonal influenza, early response strategies in the ROK (e.g., confirmation testing, social distancing) were not effectively changed because of limited national data regarding clinical characteristics.

Our study has some limitations. First, we could not include costs incurred by asymptomatic patients or those not admitted to the hospital in calculating influenza-related costs. We considered preventative actions taken by the general population and execution of the budget (including an improved response system) and its promotion to the general population. Second, the rapid antigen test for influenza was also widely used in the ROK. However, the National Health Insurance Corporation did not cover this test; therefore, we could not acquire data regarding its use or include these costs in our analysis. Third, we estimated outpatient sick leave based on the first clinic visit only and assumed no recurrence or infection with other influenza strains. Therefore, the diagnostic and productivity costs may also be underestimated. In addition, misclassification and underreporting may affect our study. When pneumonia occurs as a complication of influenza, influenza cases may be coded as pneumonia (ICD-10-CM codes: J12–J18). However, misclassification or underreporting of influenza may have been lower in the 2009–2010 season (when confirmation tests were widely used), resulting in overestimated differences in medical costs. We found that the number of pneumonia cases and fatal cases in the 2009–2010 season were similar to numbers reported in previous seasons (Table S1). Therefore, misclassification did not appear to have affected our results. Mortality data were obtained from death certificates for the 2007–2008 and 2008–2009 seasons. When several disease codes can be used as cause of death, the most severe disease is generally used on a death certificate. Therefore, influenza mortality may be misclassified/underreported, underestimating the costs of premature mortality. Because in 2009–2010 season the KCDC mortality surveillance data was used, the costs of premature mortality of 2009–2010 season could not compare with those of previous season.

Because of this potential underestimation in influenza-associated costs, the true socioeconomic burden of pandemic (H1N1) 2009 may exceed our estimate. Although a conservative formula was used, we found that total costs and average costs per visit of pandemic (H1N1) 2009 were significantly higher than those of typical seasonal influenza.

In conclusion, medical and socioeconomic costs of pandemic (H1N1) 2009 were considerably higher than costs of the previous two influenza seasons, primarily because of longer sick leave and high diagnostic costs. Therefore, government policies such as risk communication concerning sick leave and diagnostic testing could be modified to reduce the socioeconomic burden in similar situations. In addition, a surveillance system for clinical characteristics (e.g., susceptibility, treatment efficacy, adverse drug reactions, and severity) in the early stages of an outbreak is needed.

## Supporting Information

Table S1 **Annual Medical Costs for Pneumonia (ICD-10-CM: J12–J18) in the Republic of Korea, 2007–2010.**
(DOCX)

## References

[1] Dawood FS, Jain S, Finelli L, Shaw MW, Lindstrom S, et al (2009) Emergence of a novel swine-origin influenza A (H1N1) virus in humans. N Engl J Med 360: 2605–2615. 19423869 10.1056/NEJMoa0903810

[2] World Health Organization (2009) World now at the start of 2009 influenza pandemic. Geneva, Switzerland: World Health Organization.

[3] Lee J, Jeong E, Lee H (2010) Government Measures against Pandemic Influenza. J Korean Med Assoc 53: 52–58.

[4] Lee DH, Shin SS, Jun BY, Lee JK (2010) National level response to pandemic (H1N1) 2009. J Prev Med Public Health 43: 99–104. 20383041 10.3961/jpmph.2010.43.2.99

[5] Kim JH, Yoo HS, Lee JS, Lee EG, Park HK, et al (2010) The spread of pandemic H1N1 2009 by age and region and the comparison among monitoring tools. J Korean Med Sci 25: 1109–1112. 20592911 10.3346/jkms.2010.25.7.1109 PMC2890896

[6] KCDC (2009) Case of Novel Influenza A(H1N1) Infection in the Republic of Korea. Public Health Weekly Report Volume 2: 293–295.

[7] Bautista E, Chotpitayasunondh T, Gao Z, Harper SA, Shaw M, et al (2010) Clinical aspects of pandemic 2009 influenza A (H1N1) virus infection. N Engl J Med 362: 1708–1719. 20445182 10.1056/NEJMra1000449

[8] Falagas ME, Cholevas NV, Kapaskelis AM, Vouloumanou EK, Michalopoulos A, et al (2010) Epidemiological aspects of 2009 H1N1 influenza: the accumulating experience from the Northern Hemisphere. Eur J Clin Microbiol Infect Dis 29: 1327–1347. 20623384 10.1007/s10096-010-1002-3

[9] Lee JS, Shin KC, Na BK, Lee JY, Kang C, et al (2007) Influenza surveillance in Korea: establishment and first results of an epidemiological and virological surveillance scheme. Epidemiol Infect 135: 1117–1123. 17291376 10.1017/S0950268807007820 PMC2870674

[10] Lee YK, Kwon Y, Kim DW, Song KM, Cho H, et al.. (2011) 2009–2010 novel influenza A (H1N1) vaccination coverage in the Republic of Korea. Am J Infect Control. 10.1016/j.ajic.2011.05.015 21868134

[11] van Hoek AJ, Underwood A, Jit M, Miller E, Edmunds WJ (2011) The impact of pandemic influenza H1N1 on health-related quality of life: a prospective population-based study. PLoS One 6: e17030. 21399678 10.1371/journal.pone.0017030 PMC3047534

[12] Shrestha SS, Swerdlow DL, Borse RH, Prabhu VS, Finelli L, et al (2011) Estimating the burden of 2009 pandemic influenza A (H1N1) in the United States (April 2009-April 2010). Clin Infect Dis 52 Suppl 1S75–82. 21342903 10.1093/cid/ciq012

[13] Mogasale V, Barendregt J (2011) Cost-effectiveness of influenza vaccination of people aged 50–64 years in Australia: results are inconclusive. Aust N Z J Public Health 35: 180–186. 21463417 10.1111/j.1753-6405.2010.00639.x

[14] Gonzalez-Canudas J, Iglesias-Chiesa JM, Romero-Antonio Y, Chavez-Cortes C, Gay-Molina JG, et al (2011) Cost-effectiveness in the detection of influenza H1N1: clinical data versus rapid tests. Rev Panam Salud Publica 29: 1–8. 21390413

[15] Simmerman JM, Uyeki TM (2008) The burden of influenza in East and South-East Asia: a review of the English language literature. Influenza and Other Respiratory Viruses 2: 81–92. 19453467 10.1111/j.1750-2659.2008.00045.x PMC4634698

[16] Homaira N, Luby SP, Alamgir A, Islam K, Paul R, et al (2012) Influenza-associated mortality in 2009 in four sentinel sites in Bangladesh. Bull World Health Organ 90: 272–278. 22511823 10.2471/BLT.11.095653 PMC3324868

[17] Kim KS, Lee YJ (2010) Developments and general features of national health insurance in Korea. Soc Work Public Health 25: 142–157. 20391258 10.1080/19371910903547017

[18] Korean Statistical Information Service (2013) Korean Statistical Information Service. Daejeon, Republic of Korea: Korean National Statistics Office.Available: http://kostat.go.kr/portal/korea/index.action. Accessed 2013 Jun 17.

[19] Korea National Health and Nutrition Examination Survey (2006) The Third Korea National Health and Nutrition Examination Survey (KNHANES III), 2005. Seoul, Korea: Korea Centers for Disease Control and Prevention.

[20] Keech M, Beardsworth P (2008) The impact of influenza on working days lost: a review of the literature. Pharmacoeconomics 26: 911–924. 18850761 10.2165/00019053-200826110-00004

[21] Galante M, Garin O, Sicuri E, Cots F, Garcia-Altes A, et al (2012) Health services utilization, work absenteeism and costs of pandemic influenza A (H1N1) 2009 in Spain: a multicenter-longitudinal study. PLoS One 7: e31696. 22348122 10.1371/journal.pone.0031696 PMC3279412

[22] Korean National Assembly Budget office (2010) A comprehensive analysis of settlement, 2009. Seoul, Korea: Korean National Assembly Budget office.

[23] Samsung Economic Research Institute (2009) Consumer attitudes survey. Seoul, Korea: Samsung Economic Research Institute.

[24] National Health Insurance Corporation (2010) 2009 National Health Insurance Statistical Yearbook. Seoul, Korea: National Health Insurance Corporation and Health Insurance Review and Assessment Service.

[25] Mota NV, Lobo RD, Toscano CM, Pedroso de Lima AC, Souza Dias MB, et al (2011) Cost-effectiveness of sick leave policies for health care workers with influenza-like illness, Brazil, 2009. Emerg Infect Dis 17: 1421–1429. 21801619 10.3201/eid1708.101546 PMC3381579

[26] Creanga AA, Kamimoto L, Newsome K, D'Mello T, Jamieson DJ, et al (2011) Seasonal and 2009 pandemic influenza A (H1N1) virus infection during pregnancy: a population-based study of hospitalized cases. Am J Obstet Gynecol 204: S38–45. 21507375 10.1016/j.ajog.2011.02.037

