# Supplementary material for: Socioeconomic Burden of Influenza in the Republic of Korea, 2007–2010
Source: PLoS One. 2013 Dec 27;8(12):e84121. doi: 10.1371/journal.pone.0084121 (PMC3873972; doi:10.1371/journal.pone.0084121)
Supplement: Table S1 — Annual Medical Costs for Pneumonia (ICD-10-CM: J12–J18) in the Republic of Korea, 2007–2010. (DOCX) [file pone.0084121.s001.docx]

Table S1. Annual Medical Costs for Pneumonia (ICD-10-CM: J12–J18) in the Republic of Korea, 2007–2010

|  | Inpatient | | Outpatient | | Fatal cases*  (person) |
| --- | --- | --- | --- | --- | --- |
|  | Number of visits | Costs  (million US$) | Number of visits | Costs  (million US$) |  |
| 2007–2008 Season | 455,176 | 471.25 | 4,194,933 | 71.84 | 4,955 |
| 2008–2009 Season | 434,777 | 484.72 | 3,936,482 | 67.53 | 5,740 |
| 2009–2010 Season | 429,326 | 504.62 | 3,814,274 | 69.41 | 4,247 |

* The number of fatal cases for 2007–2009 was obtained from death certificates (Korean National Statistical Office). However, national death certificates for 2010 were not available; therefore, in the 2009–2010 season, fatal cases included only those occurring between May and December 2009.
